# ‘We are inheritors of a rural civilisation’: rural complexity and the ceramic economy in the Indus Civilisation in northwest India

**DOI:** 10.1080/00438243.2019.1601463

**Published:** 2019-06-18

**Authors:** Danika Parikh, Cameron A. Petrie

**Affiliations:** Department of Archaeology, University of Cambridge, Cambridge, UK

**Keywords:** Indus Civilisation, South Asia, rural, villages, complexity, ceramics, craft production

## Abstract

What role do villages play in ‘an urban civilisation’? Although it is likely that most of the populations of South Asia’s ancient Indus Civilisation would not have lived in cities, it is not clear what their rural way of life would have encompassed. Using ceramic assemblages excavated from Indus-period villages in northwest India, alongside ethnographic records on village organization and rural craft production, this paper argues that Indus villages were characterized by rural complexity. This comprised a range of activities, including craft production, as well as short- and long-distance socio-economic links. Drawing on historical narratives, we show how South Asian villages have been essentialized and presented as either ideal or conservative extremes. We argue for the importance of a better understanding of the breadth and nuances of the rural sphere, and for a greater research focus on village life in the Indus context.

## Introduction

South Asia has long been viewed as a region well suited to rural lifeways, and while this rural nature has been overly idealized, this view is also grounded in some underlying truths. Of the 717,549 settlements recorded in the 1891 *Census of India* (covering India, Pakistan, Bangladesh and Myanmar) 715,514 were classified as villages (Baines 1893, 42). Writing of India 18 years before Independence and Partition, Gandhi (1929) wrote, ‘We are inheritors of a rural civilisation. The vastness of our country, the vastness of the population, the situation and the climate of the country have, in my opinion, destined it for a rural civilisation.’ It has since been argued that it was under British colonial rule that this essentializing of India as a land of villages took place (e.g. Inden 1990, 30; Jodhka 2002, 3343), as it fed into Orientalist narratives of stagnant societies and the unchanging East, and helped justify colonial rule as an external modernizing force (e.g. Kraemer 1963, 88–89; Said 1978). This troubled history has made the task of unpicking the realities of South Asian rurality from what has historically been idealized somewhat challenging.

**Figure 1.**
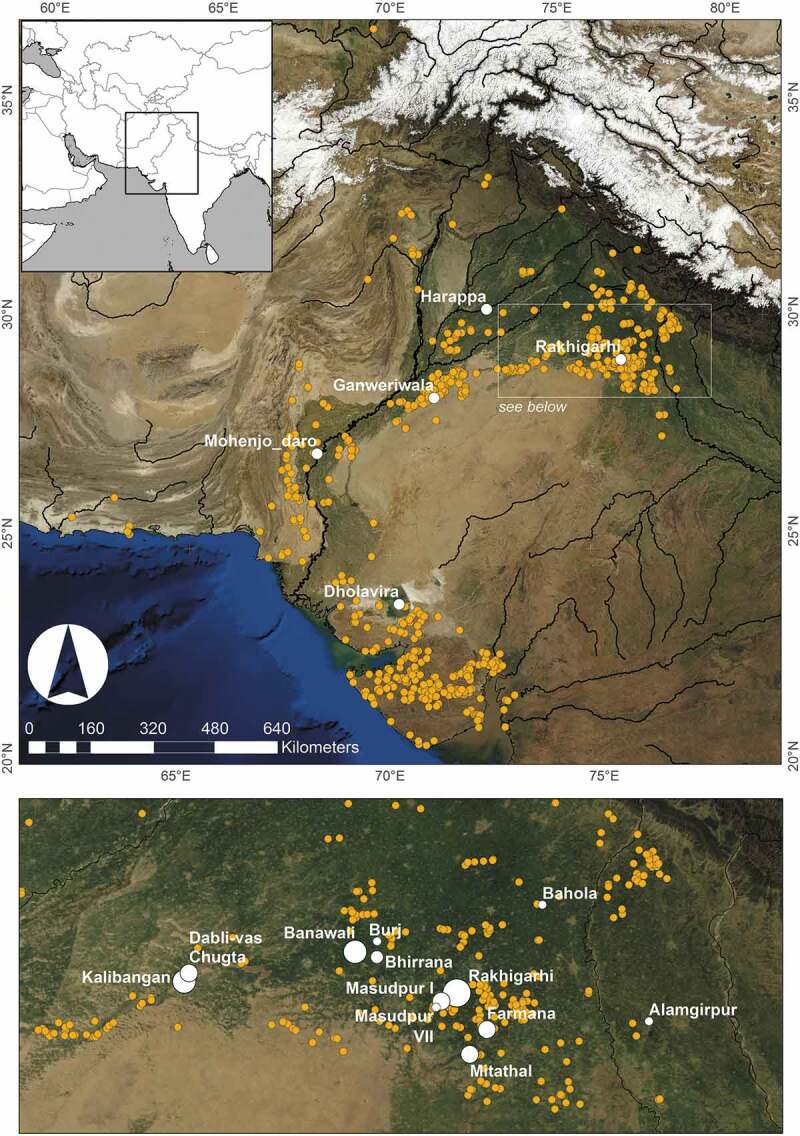
Map of the Indus cities and small sites in the text.

**Figure 4.**
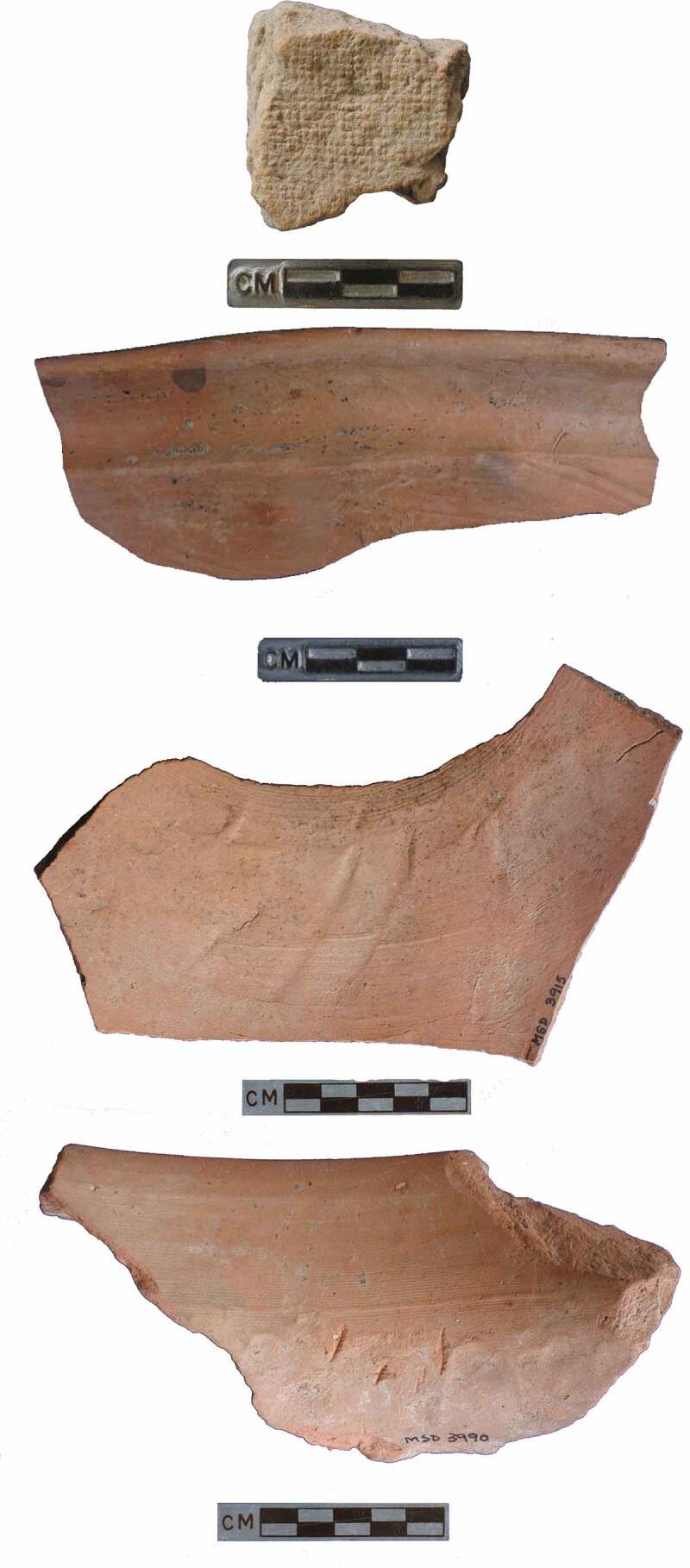
Techniques used to produce the case study village ceramics.

The first experiments with urbanism in South Asia began during the third millennium BC, when the people of what we now term the Indus Civilisation built a series of impressive cities and occupied them for an extended period (c.2600–1900 BC)  (Figure 1). From the re-discovery and excavation of the first of these cities in the early twentieth century, the Indus has been considered an urban society (e.g. Marshall 1931). Since then, large city sites such as Mohenjo-daro and Harappa have drawn a considerable amount of research, and urbanism has become such a dominant lens through which researchers have viewed the Indus Civilisation that most dating systems divide it into what are either directly or implicitly ‘Pre-urban’, ‘Urban’ and ‘Post-Urban’ chronological periods (Possehl 1977); alternatively referred to as ‘Early Harappan’ (3200–2600 BC), ‘Mature Harappan’ (2600–1900 BC) (or ‘Harappa phase’ after Kenoyer 1998, 26), and ‘Late Harappan’ periods (1900–1600 BC) (Mughal 1970; Possehl 2002; Wright 2010; contra Shaffer 1992; Kenoyer 1998). Orientalist narratives, too, have been influential, as although colonial archaeologists may have considered the Indus Civilisation urban, many also wrote of its rigid and unchanging nature (e.g. Piggott 1950, 138–141).

Despite this urban focus, the essentially rural nature of most Indus settlements has long been recognized, with Fairservis (1961, 15) noting that ‘in contrast to a multiplicity of urban sites we have a majority of village sites’. More recently, arguments about the extent to which the Indus Civilisation was urbanized (e.g. Cork 2011) and the importance of the rural sphere continue to be reiterated (e.g. Wright 2010; Petrie 2013; Petrie et al. 2017). As in modern South Asia, in the Mature Harappan period there was a contrast between relatively large urban centres and much smaller settlements. However, as we will argue, rather than an urban–rural dichotomy, this relationship was a dialectic characterized by connectivity.

Within the Indus Civilisation, only four or five cities have been discovered to date, distributed across an enormous area of modern Pakistan and India. The shortest distance between two cities was 280 km (Harappa and Ganweriwala), and the longest was 839 km (Rakhigarhi and Dholavira) (Kenoyer 1997; Petrie 2013). Kenoyer (1997, 54, 1998, 50, Table 3.1) has suggested that the geographical extent of the putative states that were controlled by these cities (‘city-states’) was between 100,000 and 170,000 km^2^, with these areas assumed to have been vast rural hinterlands. Considering the infrequency of urban sites and the distances between them, it is likely that most Indus people would have lived in small or medium-sized settlements (Petrie 2013). Madella and Fuller (2006: Figure 9) calculated that the average site size in the Mature Harappan period was 7.2 ha, which is only slightly higher than the Early Harappan average of 4.5 ha and the Late Harappan average of 3.5 ha, indicating that villages were always predominant in terms of settled area. It is now possible to use historic map analysis and remote sensing technologies that allow the locating of what may have been previously undetectable small sites (Petrie et al. 2019), more extensive and accurate surveys are emphasizing the density of the Indus rural settlements (e.g. Singh et al. 2010b, 2011; Dangi 2011; Pawar 2012; Parmar et al. 2013; Singh et al. 2018b), and overt attempts are being made to excavate smaller sites situated in the hinterlands of larger town and city sites (Petrie et al. 2017). We thus now have the tools to build on earlier intuitive insights, and to turn our sights to these smaller settlements to better understand how rural communities lived.

This paper will examine rural settlements and the relationships between the rural and the urban in the Indus Civilisation in three stages. We will first characterize rural occupation and villages in the Indus region. We will then focus on ceramic assemblages from rural Indus settlements in northwest India as a means of studying the Indus economy and the materialization of identity at Indus villages. The ceramic data being discussed come from four village sites situated in the hinterland of larger towns and cities in the northwest Indian states of Haryana and Rajasthan, that were excavated by the *Land, Water, Settlement* project (LWS). We will then examine the socio-economic role of small settlements using comparative data from other rural sites, and consider historic and ethnographic records from India to broaden our understanding of the nature of rural dynamics and networks. Building on these data and records, an argument is made for ‘rural complexity’ (Falconer 1994, 305): an acknowledgement of the multifaceted roles and variable nature of Indus rural sites, alongside an expansion of how we conceptualize these small settlements.

## Characterizing rural occupation

Most schemes proposed by scholars to classify Indus settlements are based on size. Here we rely on those proposed by Mughal (1990a) and Kenoyer (1998), who classified settlements up to 10 ha in size as villages (though Mughal distinguishes between small and large villages). We also further classify settlements between 10 and 80 ha as towns, and those greater than 80 ha as cities.

While the large size and exceptional nature of the Indus settlements that have been classified as being urban is clear, it is arguable that a better understanding of the other types of settlements is needed. Though we have classified these settlements on the basis of area, overall size is not necessarily a robust indicator of site function, and at present, the differences between Indus settlements of varying sizes is not totally clear. As Kramer (1994) noted, archaeologists must develop ‘measures of site function independent of size’, and we will demonstrate here that Indus settlements that have been classified as villages had multiple functions beyond agriculture and food production.

The variable nature of small Indus sites has been explored in different Indus regions, including notably Cholistan (Mughal [1982] 1993, 1990a, 1990b, 1994b) and Gujarat (e.g. Mehta [1982] 1993; Bhan 1994). More recently, evidence from the small sites of Bagasra and Shikarpur (25 km apart across the Gulf of Kutch at its narrowest) has been interpreted to suggest that residents of each settlement publicly identified with one another, but privately materialized social identities in different ways (Chase et al. 2014b). Here we will explore how identity is materialized at village settlements in Haryana and north Rajasthan in northwest India, and then consider variation between small Indus sites in different regions.

## Indus villages and ceramic production in northwest India

Ceramics have long been considered one of the most ‘reliable’ indicators of an Indus Civilisation settlement, particularly in the case of identifying sites through surveys, and to a lesser extent, excavation. While the regional differences between ceramics have been a feature of the research landscape for some time (e.g. Ghosh 1952; Bhan 1975; Dikshit 1984; Kenoyer 1989), it is also becoming increasingly evident that there is variation in the ceramics used at urban and rural sites within some areas (Parikh and Petrie 2017; Petrie et al. 2018). The ceramic assemblages discussed here are from four village sites excavated by the LWS project: Masudpur I (c.6 ha), Masudpur VII (c.1 ha), Burj (c.2 ha), and Dabli-vas Chugta (c.5-6 ha). Each of these sites has a specific spatial relationship to a more well-known, substantial and/or potentially urban settlement. Masudpur I and VII lie in the hinterland of Rakhigarhi, Burj is close to the site of Kunal, and relatively close to Banawali, while Dabli-vas Chugta is in the hinterland of Kalibangan. As small settlements under 9 ha in size, these villages present an opportunity to examine the lifeways of different rural Indus communities in northwest India. These villages were not all occupied in the same phases, with only Masudpur VII showing occupation during the Early, Mature, and Late Harappan periods. Burj was occupied during the Early and Mature phases, and Masudpur I and Dabli-vas Chugta were occupied during the Mature Harappan. The ceramic assemblages can provide valuable insights into rural life by indicating whether ceramic forms, technology, and decorative motifs were shared at urban and rural sites, and whether these settlements produced and consumed ceramics in the same way. Shared technologies and similar techniques at small and large settlements would suggest that the different ceramics in use at these settlements were in fact made by the same communities of practice (Lave and Wenger 1991; Wenger 1999; see Green 2016 for a case study of communities of practice among Indus seal carvers); differences would suggest separate communities of practice, with varying methods of training and potentially little contact. The first aspect of ceramic production we will consider is fabric, followed by form, then technology, and lastly surface decoration.

The ceramic assemblages from all four of these villages were made up of three fabrics. The first is a red fabric of medium texture and a limited quantity of inclusions that is particularly common across the Indus Civilisation; it shows the most variety in technique as well as decoration. The second is a thick and somewhat coarse red ware with more inclusions and voids. Many of the latter are channel-shaped, suggesting the addition of chaff as a temper. Vessels of this fabric are rarely decorated in any way. The third fabric is particular to northwest India: a grey fabric of fairly fine quality, which is distinguished by a grey-slipped and burnished exterior. A fourth buff fabric is also associated with this regional ceramic tradition (Garge 2010), although it was absent at all four LWS sites. These fabrics dominate the assemblages proportionally and have been termed Sothi-Siswal (Ghosh 1952, 37–42; Bhan 1975; Dikshit 1984, 531–537; Bala 2003) or Haryana Harappan ceramics (Parikh and Petrie 2017). No sherds of the Classic Harappan ceramics typically found at larger sites have been documented.

**Figure 3.**
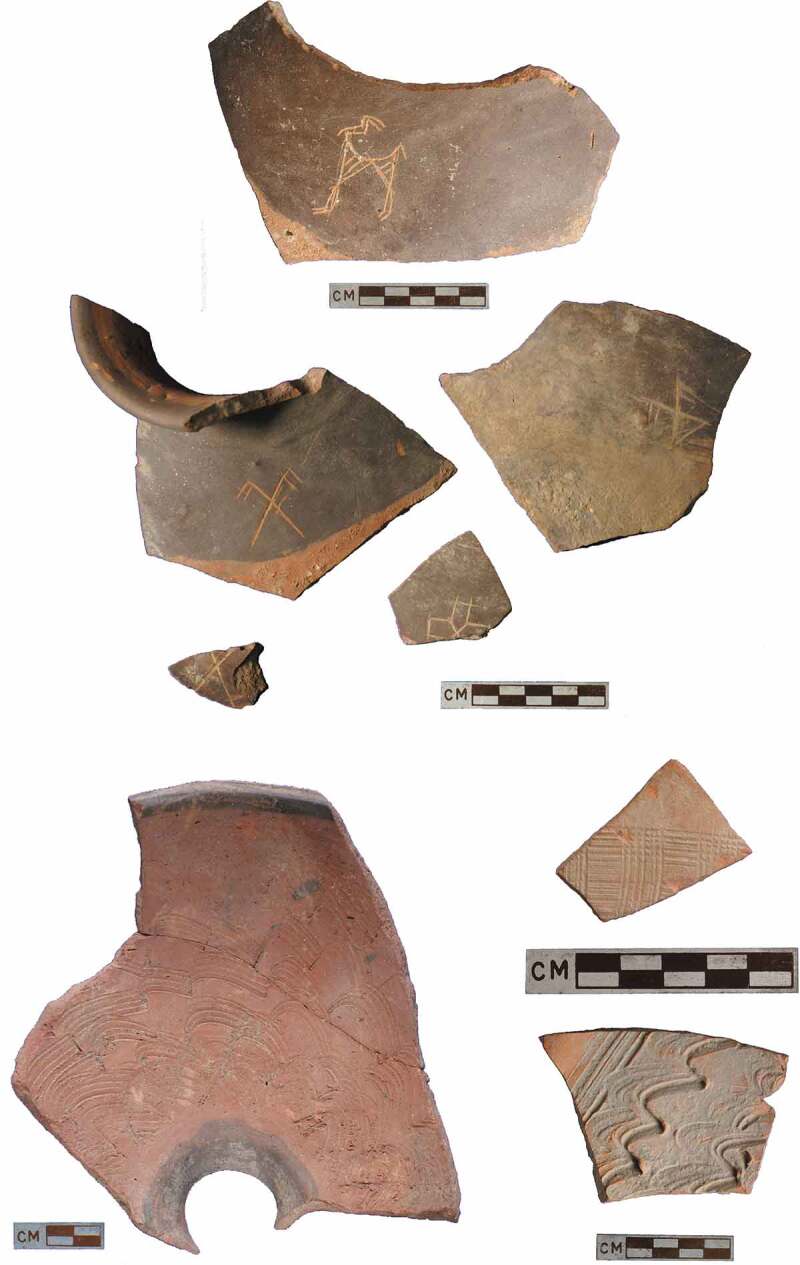
Incised motifs of the case study village ceramics.

The Classic Harappan ceramics that are characteristic of the urban phase have distinctive vessel forms, and show the predominant use of the potter’s wheel to form the vessels, and specific approaches to surface decoration, including a dark-red slip and a particular repertoire of decorative motifs (Dales and Kenoyer 1986; Jenkins 1994; Quivron 2000). Although some Classic Harappan forms found at urban settlements, such as large ornately painted jars, are absent from the four village sites that we have examined, other vessel forms including Harappan-style cooking pots, perforated jars, and dish-on-stands are present, made in the local fabrics. It is clear that there is a long tradition of region-specific forms, however, and vessel types such as basins incised with decorative motifs on the interior, as recorded in the Early Harappan phases at Kalibangan (Thapar 1975; Bala 2003), are specific to northwest India; they are also common at the LWS village sites. It is also notable that some vessel forms and decorative motifs that we have observed have not been recorded at any other Indus settlements, such as unusual incised and painted funnels from the Early Harappan deposits at Burj (Figure 3). During the Mature Harappan period, vessel forms are where the most overlap between the Classic and Haryana Harappan ceramic assemblages can be observed, whereas greater variation is observed in decorative treatments and technology.

In terms of technology, a variety of techniques were in use at the LWS sites, many in conjunction with the potter’s wheel, and we have observed a fluidity in the techniques used to produce the same vessel. Despite the introduction of the potter’s wheel, potters continued to create Haryana Harappan ceramics using other techniques like hand and coil-building. Working with the potter’s wheel requires a long apprenticeship (Roux and Corbetta 1990) and its infrequent use in the production of rural ceramics in the Indus context has interesting implications for connections *between* communities of practice, as well as the choices of individual potters. Perhaps most interestingly, rural potters explored complex *chaînes opératoire* that included the use of multiple techniques to produce a single vessel, such as coil-making and wheel-finishing. While at this point we have only studied surface traces to determine the techniques used, analysis and experimental work on surface traces and microfabrics of ceramics from Kalibangan reinforces the suggestion that coil-building, wheel-finishing, and a variety of wheel-fashioning methods were used in the region (Courty and Roux 1995; Roux and Courty 1998). Five sherds from Masudpur VII also showed clear evidence of textile impressions, suggesting they had been draped with textiles prior to firing. Although textile impressions on ceramics are relatively uncommon in the Indus Civilisation, they have been previously recorded at other sites, including Harappa, where jute was identified (Wright et al. 2012). We have noted the use of the wheel to produce or finish rims but not the vessel body, as well as visible join marks showing where rims were attached to bodies and then wheel finished (Figure 4). There is also clear evidence for the use of extensive scraping and smoothing that in many cases may have obliterated evidence of all previous techniques used.

**Figure 5.**
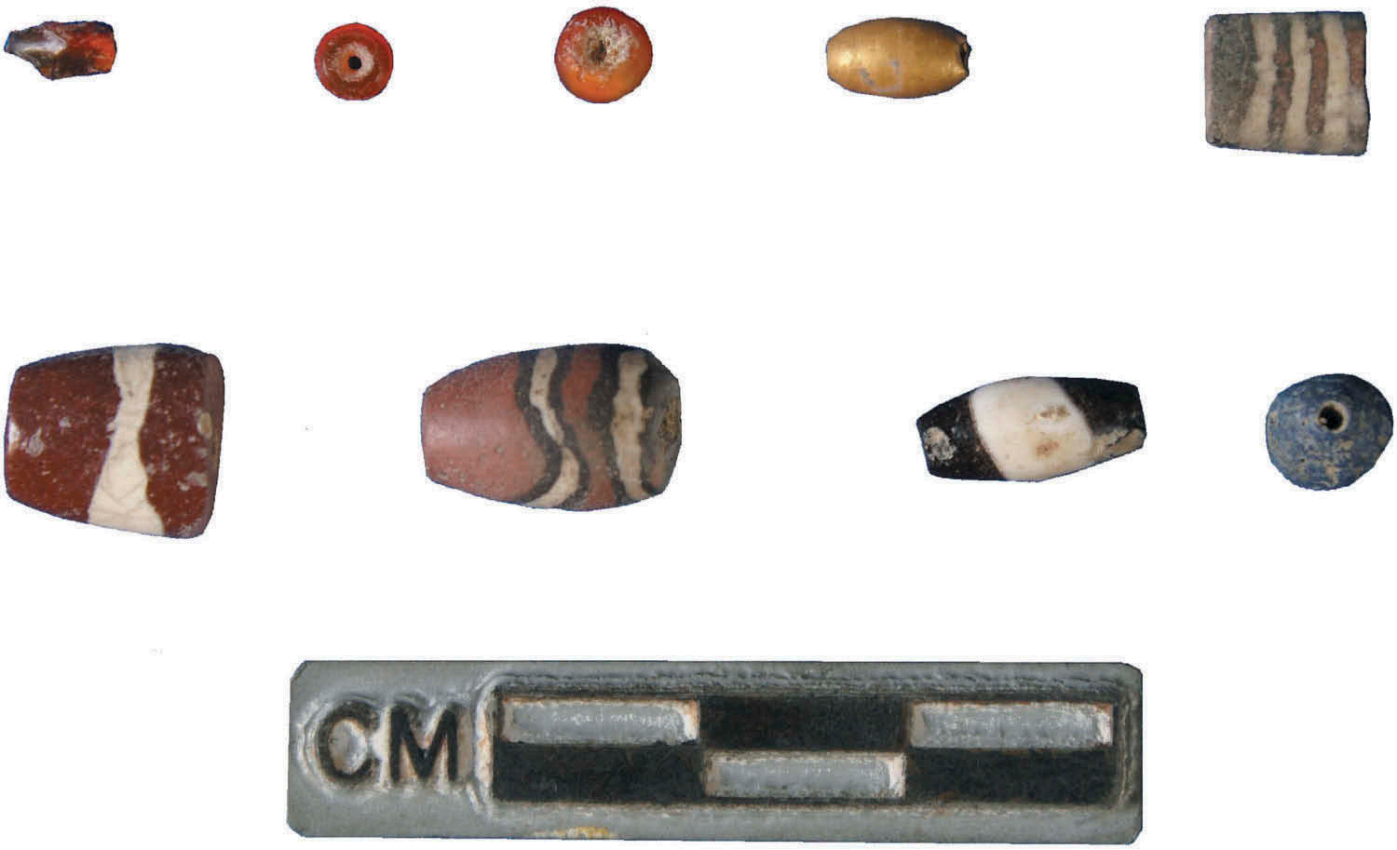
Agate, lapis lazuli, gold and carnelian beads from Masudpur I and Masudpur VII.

**Figure 2.**
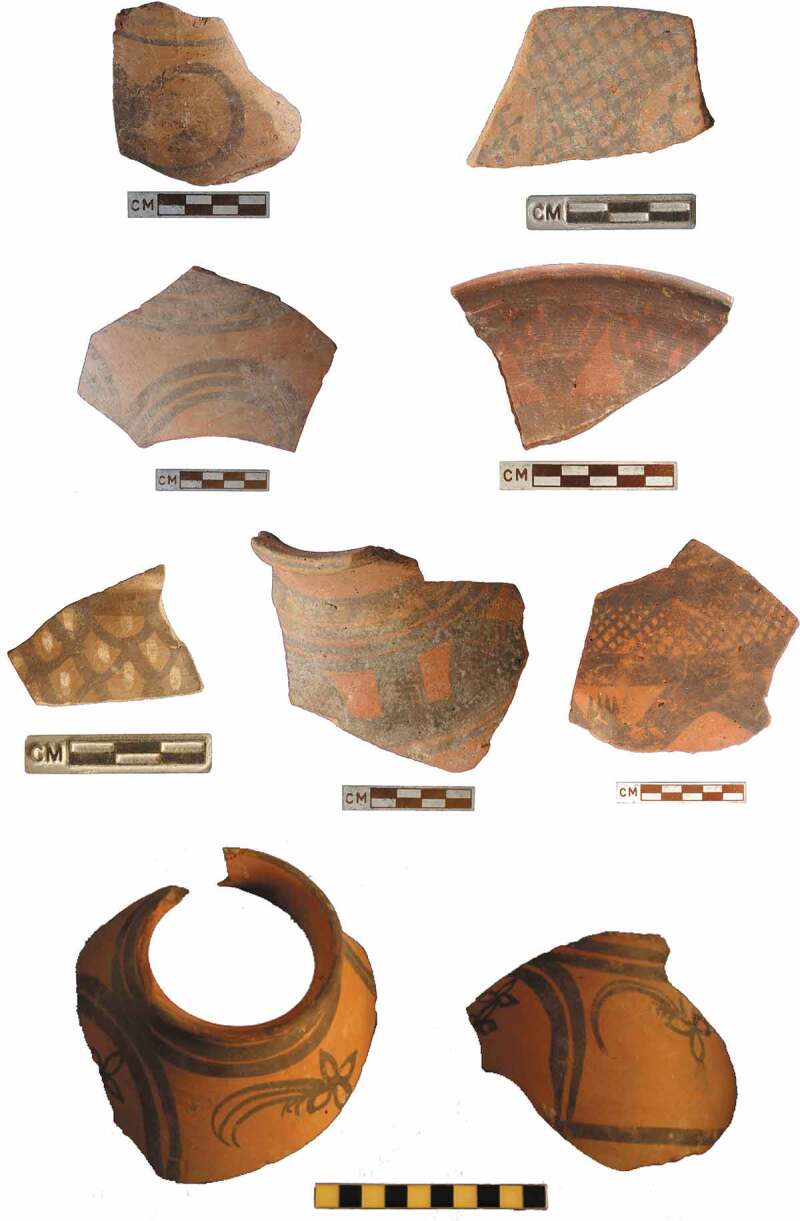
Painted motifs of the case study village ceramics.

Surface decoration is where the Haryana Harappan ceramics differ most from the Classic Harappan, and again this variation begins in the Early Harappan and continues into the early phases of the Mature Harappan periods. At the LWS sites, some red ware vessels were decorated with both red and brown slips, the latter occasionally burnished to a deep glossy finish, and others were painted with black/brown and white pigments, which is a regionally distinct preference. These two colours were used to create what is referred to locally as bichrome pottery and that in conjunction with a set of regionally distinct motifs resulted in vessels quite clearly visually different from ceramics commonly used elsewhere. Pre-firing incised decoration is common in northwest India, where it is typically referred to as Kalibangan Fabric D (Thapar 1975; Bala 2003), and it was recorded at all four of these LWS village sites, even if motifs varied. In terms of painted motifs, there was a preference for bands, wavy lines, and relatively simple geometric motifs at Masudpur I, Masudpur VII and Burj, but at Dabli-vas Chugta we also observed nature-inspired motifs including floral themes that are not common on Indus pottery of this period, though have previously been reported from the nearby site of Kalibangan (Bala 2003, 103, Figures 19, 21, 43) (Figure 2). None of the highly distinctive Classic Harappan decorative motifs such as pipal leaves and intersecting circles are seen at these sites, but importantly, Haryana Harappan ceramics were found at Rakhigarhi – as we observed when examining material from the original excavations (Nath 2015). Our research at sites in northwest India thus suggests that in many ways a different visual vocabulary was used at smaller settlements. Crucially, the motifs observed on the ceramic vessels are often the only forms of iconography excavated at these villages, and the potential that different motifs were deliberately used to produce ceramics that were visually distinct from those used in other regions should not be overlooked.

There is thus long-term variability in the form, technology and decoration of Indus-period ceramics. The Early Harappan ceramics from Masudpur VII and Burj are broadly similar and have much in common with the Sothi-Siswal types seen elsewhere in northwest India. Nevertheless, there is clear variation between the ceramic finds at these sites, which are about 50 km apart, and this material is clearly different to contemporaneous ceramics found in other parts of the greater Indus region. The Mature Harappan ceramic assemblages from Masudpur I, Masudpur VII and Dabli-vas Chugta are again broadly related, but distinct from one another and material seen elsewhere, showing both spatial and temporal variation in vessel forms and decorative styles. The regional rural ceramic economy in northwest India was clearly complex and shows a considerable degree of variation. Rural communities produced some ceramic forms similar to Classic Harappan forms, and others that were quite different, and they used some decorative motifs that were common and others that we had previously not seen. This pattern of similar ceramic forms but different techniques and decoration is particularly interesting, given what we understand of how pottery production is learned. Pottery forming is often learned through ‘vertical transmission’, inter-generationally; shape and decorative motifs are more easily imitated and are often transmitted horizontally, or peer-to-peer (Knappett 2011, 106–107; see also Gosselain 2000). The use of different techniques to produce the same forms suggests that Classic Harappan and Haryana Harappan ceramic material was not produced in the same workshops, and that these potters are unlikely to have been members of the same communities of practice.

Beyond the ceramic material, comparable patterns of similarities and differences between village and urban contexts may be detected in other iconographies, including small terracotta animal figurines, only some of which are similar to those found at Harappa and other urban sites. A single small red-slipped, burnished, and painted pinched-face anthropomorphic figurine found at Masudpur VII (Petrie, Singh, and Singh 2009) bears little resemblance to Indus figurines seen at urban settlements, and other figurines of this type have not been recorded at any of the Indus cities or larger settlements. Interestingly, a similar small figurine was excavated at Nagwada, although this was poorly made and had the addition of some micro-bead inlay (Hegde et al. 1990). That the only iconographic material and representations of humans at several small settlements look distinct from those seen at urban centres could have profound implications for our understanding of how rural communities may have self-identified.

In addition to the figurines, other material culture from these sites is seemingly at odds with the local or regional-dominated ceramic assemblages. Much of it comprises what might be described as a ‘typical’ Indus assemblage of steatite micro-beads, steatite disc beads, faience and terracotta bangles, terracotta ‘cakes’, and beads of various semi-precious and precious stones and metals, including agate, carnelian, lapis lazuli and even gold (e.g. Petrie, Singh, and Singh 2009; Singh et al. 2010a, 2012) (Figure 5). While the terracotta objects are likely to have been made from locally available raw materials, the semi-precious stones and metals were certainly not locally available (see Law 2011). The small artefacts from these sites thus demonstrate how these villages were embedded in wider Indus exchange and trade networks, with access to what have long been thought of as luxury items made from raw materials sourced from hundreds of kilometres away.

Taken together, this evidence suggests that the ceramics seen in the rural context are related to those seen in the neighbouring urban centre, but many ceramics seen at the urban centre are not seen in the hinterlands and vice versa. It is possible that villagers did not have access to the Classic Harappan ceramics seen in the urban context, but given they were able to access a range of other potentially more elite objects, it is not clear why ceramics would have been restricted. This pattern suggests that despite the geographic proximity and presumable socio-economic connections between urban centres and rural settlements in their hinterlands, the rural populations may have practised some amount of creative independence. It also suggests the existence of distinct communities of practice that produced these rural ceramics.

## The socio-economic role of small settlements

Given the substantial distances between the Indus cities and large sites, the role of these ‘nodes’ and the nature of settlement in the intervening areas is crucial to a fuller understanding of Indus societies. Many small settlements duplicate features of larger Indus settlements such as fortifications, water systems, and planned layouts, but the function of these settlements is not clearly understood (Mughal 1994a, 54). Chakrabarti (1995, 116) has suggested that ‘the distinction between a village, a town and a city is to some extent blurred among the Harappan settlements’, arguing that the size of a settlement seems to bear no relationship to the level of its planning and craft practices. Excavations at Allahdino, a 4 ha site in Sindh, revealed covered house drains, stone drains, and what appears to be an irrigation system (Fairservis 1979, 99, [1982] 1993). It has been described as a ‘fully developed agricultural village with an irrigation channel, a household water system and some public buildings’ (Eltsov 2008, 96; Hoffman 1974).

Small settlements as foci of highly specialized craft production are not uncommon in the Indus Civilisation, and several have been referred to as factory sites (e.g. Vidale 2000, 38). It has also been suggested that medium and large ‘town-sized’ settlements played an independent rather than subordinate role that was important in both interactive processes and socio-economic control structures (Petrie 2013, 91, 94–95; Sinopoli 2015, 322; Petrie et al. 2017). Evidence suggests that some crafts were practised in rural areas and that products were taken to urban centres from these locations. Rural craft production workshops have been found at Dubi, the Veesar Valley, in the Lower Indus near Mohenjo-daro, in Cholistan, and at sites along the Beas near Harappa (Wright 2010, 332). There is plentiful evidence for access to elite crafts at smaller settlements, including at some very small settlements that may have been occupied temporarily. For example, gold beads and two hoards totalling over 30,000 steatite microbeads were recovered from the temporary site of Zekhada in Gujarat (also known as Zekda or Jekhada) (Hegde, Karanth, and Sychanthavong [1982] 1993). At Nagwada, finished lapis lazuli beads and gold ornaments were found buried beneath a floor; the site also had evidence for bead and shell production (Hegde et al. 1988; Bhan 1994). Similarly, there is ample evidence for specialized production at small settlements, such as that for bead production at Chanhu-daro, which was c.5 ha (Possehl 2002, 74), and shell bangle production at Bagasra, which was a fortified settlement 1.92 ha in size (Bhan et al. 2005). In addition to Bagasra, there are numerous small settlements with monumental walls in Gujarat (Chase et al. 2014a, 1), which appear to have served multiple purposes. Indus stamp seals, which may have had an administrative function, are also found at small sites in small quantities. Generally made from fired steatite, carved with animals and occasionally human or narrative scenes, these seals provide most of our knowledge of the as-yet untranslated Indus script. Recent research suggests that one of their purposes was to control and record access to storage containers and rooms (Frenez and Tosi 2005). At Shikarpur a steatite seal and multiple sealings with script have been found, and five steatite Indus seals and multiple sealings with Indus script were found at Bagasra (Chase et al. 2014b, 66). It is not clear how the inhabitants of these settlements were fed; they may have produced their own food, or, as ‘specialist traders and craftspeople’ they may not have had to (Chase et al. 2014a, 3). It has been proposed that the ‘infrastructure of Harappan craft production encouraged the establishment of “villages” of specialized producers, such as… Chanhu Daro…and Nageshwar’ (Bhan, Vidale, and Kenoyer 2002, 260).

Evidence thus suggests that small settlements in the Indus region were not solely agro-pastoral, but that there were farming villages, villages where craft production took place, and (perhaps most likely) villages that combined these and other activities. The site of Nausharo on the Kachi Plain in Pakistan was originally thought to be a large rural centre (Jarrige 1974–1986, 119), but was later described by the excavators as ‘a true city’ (Jarrige 1995–1996, 4). Despite being only 6 ha in size, detailed excavation revealed evidence for enough complexity that it was thought to qualify as an urban centre. While Nausharo is an unusual Indus site, it demonstrates the uneasy relationship between concepts of size, rurality, and agro-pastoralism when classifying sites. While these categories may overlap in varying ways, size does not indicate a primarily agro-pastoral settlement, and a rural settlement may show evidence for elite goods, manufacturing, socio-economic complexity, and links with both rural and urban settlements.

Building on Eltsov’s (2008) work exploring comparative evidence for authority, Table 1 explores evidence for rural complexity, i.e. complexity at Indus settlements less than 9 ha in size, including the four case study villages. The aim of this comparison is to show the range of complex economic behaviour attested at small sites in different regions of the Indus Civilisation. It is not an exhaustive comparison, nor should it be taken to suggest that evidence from one area finds direct parallels elsewhere. It should be pointed out, however, that although some of these small settlements show evidence for fortifications, as mentioned above, a number of them are located in Gujarat where it is clear that fortified settlements are far more common than elsewhere. The regionalism demonstrated during the Mature Harappan period across the Indus zone must be taken into account when thinking about small settlements and modelling rural complexity. Moreover, while this table records for instance the presence of fortifications, the nature of these may vary considerably, or in the case of a site like Chanhu-daro, be disputed. Although many small settlements may have evidence for elite goods like beads of gold, silver, or semi-precious stone (including carnelian, agate, and lapis lazuli), these may be few in number, as at Bagasra where there were significantly fewer of these compared to steatite beads (Chase et al. 2014b, 69). Additionally, in some cases evidence for manufacturing is minimal, as at Surkotada, where out of 1050 beads, only 11 unfinished agate beads were found (Joshi 1990, 310–337), or at Allahdino, where copper slag was found in limited amounts, but no furnace has yet been excavated (Fairservis [1982] 1993, 112). Vidale (2000, 44) has suggested that the small amounts of unfinished beads of semi-precious stones found at many excavated Indus sites may ‘be the result of the episodic work of part-time craft specialists living outside the cities’. Whether part time or full time, it is clear that specialized craft production in the Indus was carried out at many small settlements.

**Table 1.** Small Indus Civilisation sites with evidence for socio-economic complexity.

Region	Site	Size in ha	Fortifications	Sealsor Sealings	Classic Harappan Ceramics	Regional Harappan Ceramics	Evidence for Manufacturing	Objects of precious metal and/or semi-precious stone
Baluchistan	Nausharo	6	X	X	X	X	X	X
Sindh	Allahdino	1.40		X	X	?	X	X
	Chanhu-daro	c.5 (+?)	X?	X	X	?	X	X
Gujarat	Bagasra	1.92	X	X	X	X	X	X
	Nagwada	1.54		X	X	X	X	X
	Shikarpur	5	X	X	X	X	X	X
	Surkotada	2–3.5	X	X	X	?	X?	X
	Zekhada	4.5			X	X	X	X
Haryana	Burj	c.2				X		X
	Masudpur I	c.6				X		X
	Masudpur VII	c.1				X		X
Rajasthan	Dabli-vas Chugta	c.5–6				X		

## Rural socio-economic networks and dynamics: an ethnographic perspective

Having characterized the rural settlements of the Indus, considered the relationship between urban and rural ceramics, and discussed the economic complexity of smaller Indus sites, we now turn to ethnographic and historical perspectives. We suggest that these analogies can be used as tools to explore complexity in village life, and how ceramic production and distribution may have been integrated in urban–rural networks of the Indus. Although we do not propose direct analogues between the twenty-first century BC and the twenty-first century AD, these records will aid in our understanding of the potential breadth of socio-economic complexity in these villages, which has almost certainly been previously underestimated.

In her seminal anthropological study of ceramics in Rajasthan, Kramer (1997) wrote about village–city interactions. She noted a variety of different economic behaviours: some rural vendors spent large parts of the year in cities, making their own pottery as well as sourcing it from villages – including their own – to sell in the city, and selling this rural pottery on to other villages (Kramer 1997, 129–130). In this scenario, rural agents use an urban centre to collect and redistribute pottery. In other scenarios, however, this redistribution happens independently of a city, with villagers buying pottery from itinerant potters, or with vendors travelling large distances to villages to buy pottery to re-sell in other villages. In many cases, kinship groups and networks played a role, with marriage contributing to the movement of ‘exotic’ or unusual pottery between villages (Kramer 1997, 130).

Rural potters have long described making vessels for their own local community, as well as selling them to vendors who in turn took them to other villages (e.g. G. Singh 2015). Most recent studies in north India have shown that the market for rural potters is confined to villages within 10 km of the potter, while urban potters may supply settlements up to 200 km, or in some cases 400 km, away (Varma and Menon 2017, 23). This is not dissimilar to the 10–15 km suggested by Kramer (1982) as typical for daily or short-term interactions between inhabitants of different types of settlements, based on ethnographic work on rural settlements in Iran. These observations provide helpful models for gaining insight into the distribution of ceramics in the Indus context, and how different regional styles may have spread and reached cities and other villages in Indus-period Haryana and Rajasthan. It has been suggested that during the Indus period, some rural settlements may have had direct links with urban centres, while others may have had relatively little contact (Meadow and Kenoyer 2005), and this has subsequently been interpreted as evidence of shifting economic and political networks (Kenoyer 2008).

Ethnographic work in Gujarat in the 1960s suggests that rural potters did not cluster together, to ensure that each was able to make a living. One potter, having been trained by his uncle, left to work in another village, saying, ‘if two potters stay in one village, neither of them is able to earn enough’ (Fischer and Shah 1970, 117). In spite of this, theirs is not a solitary existence as they build small family-based workshops (Fischer and Shah 1970, 117–8). Modern ceramic production in northwest India has often taken place in what Sinopoli (1998, 163) has called ‘specialist workshops organized at the household level’, which she identified at medieval Vijayanagara in South India (AD 1340–1565). Case studies also demonstrate that professional potters in north India may have to have a second occupation, depending on whether they are based in villages, towns, or cities, which conforms to discussions of the degree to which producers were part-time or full-time specialists (see Costin 1991, 2001). Those in villages engage in pottery production during the hot and dry season, and the festive season, but engage in other work for the rest of the year (Varma and Menon 2017, 5). The monsoon season necessitates a long break from ceramic production, which further complicates the issue of how to categorize full-time specialists. Thus, ceramic specialization can vary in urban and rural contexts, and may be more fluid in villages.

The ways in which rural ceramic production were carried out across the Indus Civilisation are certainly not clear. Although no kilns or evidence of production have been found at Masudpur I, Masudpur VII, Burj or Dabli-vas Chugta, kilns were documented at the neighbouring site of Masudpur V (Petrie, Singh, and Singh 2009), and evidence for ceramic production has been seen at other smaller sites in the hinterland of Rakhigarhi, such as Lohari Ragho II (Garge 2006; Singh et al. 2018a). The variation in production techniques and decorative motifs suggests that ceramic production was diffused rather than centralized, and it appears that Classic Harappan and Haryana Harappan ceramic material was produced in different workshops, and by members of different communities of practice.

The nineteenth-century comparative jurist and legal anthropologist Maine (1871, 117) wrote ‘Nothing can be more complex than the customs of an Indian village’.^1^ While life in an Indus village in northwest India would likely not have been a direct microcosm of life in a city, rural communities appear to have had the benefit of many of the same technological developments and trade networks as their urban counterparts, though no doubt less intensively. They were active producers and consumers of a range of products, growing and processing crops (e.g. Bates, Singh, and Petrie 2017) as well as producing other commodities and in turn acquiring through exchange or other means of distribution objects of shell, semi-precious stones, and metals, precious and otherwise (Petrie, Singh, and Singh 2009). Many of these object types have long *chaînes opératoires* involving the retrieval of materials from often distant sources, treating those raw materials in specific ways (for example, carnelian must be fire-treated to achieve its signature fiery orange colour; Kenoyer, Vidale, and Bhan 1991; Kenoyer 1998; Vidale 2000), and then manufacturing the object, frequently using labour-intensive technologies (Vidale 2000; Vidale and Miller 2000).

Evidence suggests that there was a network of socio-economic connections and dynamics that would have brought different rural populations in contact with elite goods from nearby urban centres, as well as those from further afield. The spread of regional decorative styles such as the red ware with incised decoration that has been found at Masudpur I, Masudpur VII, Burj, and Dabli-vas Chugta, and its continued popularity into the Mature Harappan phase when regional ceramic styles are believed to have become less common, is indicative of the economic links between settlements of different sizes across northwest India. Ethnographic data suggests rural potters could have traded their wares to neighbouring villages, towns, and cities, and rural communities may have been able to acquire pots from non-local as well as local potters. There are thus a number of ways in which rural ceramic production could have been organized, and this is supported by evidence that this could greatly vary between settlements and regions in the Indus Civilisation.

## Conclusions

Research at villages has shown the diversity of Indus material culture, as well given us a better understanding of the regionalism that underpinned the apparent uniformity of some types of Mature Harappan material, which has been referred to elsewhere as a veneer (Meadow and Kenoyer 1997; Petrie 2013; Petrie et al. 2018). Most ‘Harappans’ would not have lived in ‘Harappas’, and the vast majority of the population is likely to have lived in smaller settlements. Given this, and the part that small rural sites have played in major developments of our understanding of the Indus, from our knowledge of regionalism to new dates for crop domestication, these sites demand further study. The relationship between villages and the towns and cities around them appears to have been complex, and both the links that connected them as well as the social boundaries that divided them bear examination. So far, the excavation of small settlements has proved enormously fruitful, even if excavating such sites is regarded as less prestigious work. To date, excavations at the LWS villages have, among other things, yielded rare or otherwise unique material culture, and have even provided evidence for early cultivation of locally domesticated rice (Petrie et al. 2016; Bates, Petrie, and Singh 2017). The rural economy is clearly more diverse than previously thought and assumed.

In 1929, Gandhi believed that India’s past and future lay in its villages. By 1945 however, he wrote to Jawaharlal Nehru, ‘My ideal village still exists only in my imagination’ (Gandhi 1989–94). Perhaps even in a rural civilization, there is no one ‘ideal village’, but a multitude of villages of different sizes and functions, developed organically, playing different roles for their inhabitants and those of other settlements both nearby and afar. We must also keep in mind that social complexity at rural settlements has many forms, and can take an ugly turn. The village *jajmani* system of caste-based hereditary specialization in India (Wiser 1936) is complex and rigidly hierarchical, as well as deeply exploitative (e.g. Chaudhry 2013). As Jodhka (2002, 3343) has pointed out, ‘for Gandhi the village was a site of authenticity…for [B.R.] Ambedkar the village was the site of oppression.’ Rather than idealize or oversimplify, it is important to study and represent the rural experience in all its breadth. We must move towards an understanding of South Asian rurality that acknowledges the diversity of the rural base, while steering clear of colonial and historic oversimplification of rural lifeways. In the search to construct an idea of national character, conducted by both British colonizers and later Indian nationalists, the commonalities of rural experiences were overstated to help build narratives for political purposes (Jodhka 2002). However, Indus and later Indian villages may have shown considerable variation, and rather than being contained, they are in fact likely to have been significantly more connected with the wider Indus world than has previously been thought.

The urban–rural dialectic (Schwartz and Falconer 1994) is more complex than a straightforward relationship where villages supplied food and consumed urban goods in return. Rural settlements present a varied array of evidence and appear to have had complex and diverse relationships with urban centres. Villages were also susceptible to change, and as seen with the larger settlements at the end of the Mature Harappan period, many Indus villages were abandoned after the Late Harappan period. Instead of seeing a simple increase in the number of villages in the Late Harappan and post-urban periods when the cities were abandoned, surveys show that villages were depopulated in some areas and new villages were established (Singh et al. 2011; Green and Petrie 2018). Many of these settlements were subsequently abandoned, as illustrated by the fact that settlement numbers in the hinterland of Rakhigarhi shrink from 32 Late Harappan sites to 18 in the later Painted Grey Ware phase (Singh et al. 2010b). As Kramer (1994) has pointed out, ‘Rural settlements may be economically (comparatively) self-sufficient, but they are not isolated’. While these village communities may have been agriculturally self-sufficient, and may potentially have self-identified as different from urban populations, their livelihood and the survival of their settlements were ultimately inextricably linked with the people living throughout the rest of the Indus Civilisation.

## Note

1. Quote in its entirety: ‘The truth is, that nothing can be more complex than the customs of an Indian village, though in a sense they are only binding on chiefs of families.’
